# A new computational method to split large biochemical networks into coherent subnets

**DOI:** 10.1186/1752-0509-5-25

**Published:** 2011-02-07

**Authors:** Wynand S Verwoerd

**Affiliations:** 1 Centre for Advanced Computational Solutions, Dept WF & Molecular Bioscience, Lincoln University, Ellesmere Junction Road, Christchurch, New Zealand

## Abstract

**Background:**

Compared to more general networks, biochemical networks have some special features: while generally sparse, there are a small number of highly connected metabolite nodes; and metabolite nodes can also be divided into two classes: internal nodes with associated mass balance constraints and external ones without. Based on these features, reclassifying selected internal nodes (separators) to external ones can be used to divide a large complex metabolic network into simpler subnetworks. Selection of separators based on node connectivity is commonly used but affords little detailed control and tends to produce excessive fragmentation.

The method proposed here (Netsplitter) allows the user to control separator selection. It combines local connection degree partitioning with global connectivity derived from random walks on the network, to produce a more even distribution of subnetwork sizes. Partitioning is performed progressively and the interactive visual matrix presentation used allows the user considerable control over the process, while incorporating special strategies to maintain the network integrity and minimise the information loss due to partitioning.

**Results:**

Partitioning of a genome scale network of 1348 metabolites and 1468 reactions for *Arabidopsis thaliana *encapsulates 66% of the network into 10 medium sized subnets. Applied to the flavonoid subnetwork extracted in this way, it is shown that Netsplitter separates this naturally into four subnets with recognisable functionality, namely synthesis of lignin precursors, flavonoids, coumarin and benzenoids. A quantitative quality measure called *efficacy *is constructed and shows that the new method gives improved partitioning for several metabolic networks, including bacterial, plant and mammal species.

**Conclusions:**

For the examples studied the Netsplitter method is a considerable improvement on the performance of connection degree partitioning, giving a better balance of subnet sizes with the removal of fewer mass balance constraints. In addition, the user can interactively control which metabolite nodes are selected for cutting and when to stop further partitioning as the desired granularity has been reached. Finally, the blocking transformation at the heart of the procedure provides a powerful visual display of network structure that may be useful for its exploration independent of whether partitioning is required.

## Background

The genome scale metabolic network of small molecule reactions for cells (particularly eukaryotic cells) is sufficiently complex that it is hard to visualize, let alone interpret. Using conventional biochemical pathways is a bottom-up approach that helps to bridge the complexity gap between individual reactions and the complete network. But this still leaves scope for an intermediate level of granularity, namely subnets. A subnet allows the study of the interplay between pathways and reactions in a broader context, while still focussing attention on a limited biological functionality of interest.

This line of thought has been pursued by many authors in the recent literature, together with algorithms that use a top-down approach utilising the inherent structure of the complete network to determine its natural subdivision points. In addition to the conceptual argument, there are also practical considerations that motivate this endeavour in particular contexts. The use of structural analysis tools such as elementary modes and extreme pathways [1], suffers from the problem of a combinatorial explosion [2] of the number of such modes in a complex network. In essence the problem is that if two small networks are joined together sequentially, each pathway in one can be joined to each pathway in the other. So reversing this and partitioning a large network into subnets is a useful strategy to keep mode numbers manageable. Alternatively, significant advances have also been made in large scale mode calculations in genome scale networks [3] and analyzing the results by sorting [4] or pattern matching [5] techniques. Whether such methods or partitioning is preferable depends on the goals of a particular project.

Another significant context is flux balance analysis (FBA). There, knowledge of at least some measured fluxes is needed in order to calculate others by applying stoichiometric and other constraints. Current technology allows simultaneous measurement of about a dozen flux values or several hundred metabolite concentrations [6]. Optimization of an objective function such as biomass production has been used successfully to supplement the constraints for metabolic modelling of unicellular organisms, but the choice of objective for multicellular organisms is problematic and even for unicellular systems, maximising biomass is not always appropriate [7]. So in a study that focuses on a particular aspect of metabolism, it would be helpful if a way can be found to limit the FBA calculation to a "relevant" section of the network and avoid needing boundary conditions that only affect other metabolic aspects.

Depending on the priority allocated to these three sets of considerations, different approaches have been advocated, and a recent review including the application of more general network theory approaches to biological networks, can be found in a recent article by Nayak and De [8].

The conceptual network simplification problem is typically addressed by clustering- or community finding algorithms. A typical example is the Markov clustering (MCL) algorithm[9]. There, the focus is on identifying groups of nodes that are closely connected to each other, while intergroup connections are weaker by comparison. A clustering method somewhat similar to MCL has also been applied to metabolic networks [10]. This approach uses simulated annealing to process connectivity information to find modules with high connectivity within and minimal links between modules, but takes no account of mass balance constraints.

However, clustering of this kind is not really appropriate for metabolic subnetworks. The most highly connected metabolites are commodity or currency compounds such as H_2_O and NADH, but generally (depending on the context) they are of least interest in terms of function. Conversely, the conventional pathways of biochemistry that should form the core of a functionally oriented partitioning are typically linear or circular and only weakly connected in terms of graph structure.

An alternative approach to the conceptual clarification of biochemical network structure is as hierarchy trees, an approach advocated in the work of Holme, Huss and Jeong [11] and of Gagneur, Jackson and Casari [12]. However, such hierarchies are not very amenable to isolating a particular subnetwork for FBA or mode analysis.

An approach that prioritises the appropriateness of a biochemical subnet for use in practical applications, was demonstrated by Schuster et al [13]. The approach is based on the observation that metabolite nodes in a biochemical network are of two distinct types: Internal nodes that have associated stoichiometric mass balance constraints, and external nodes that represent metabolite inflows and outflows from the environment and have no associated mass conservation. The external nodes define the periphery of the network, and so a new boundary that separates a subset of nodes from the rest can be created by reclassifying some of the internal nodes as external. Changing the status of a node from internal to external, can in a graphical network representation be seen as splitting it into two: one copy becomes a sink in one subnet, and the other a source in another subnet. This demonstrates another difference from clustering, where networks are usually partitioned by deleting links rather than splitting nodes. The selection criterion used [13], is that all internal metabolites that participate in more reactions than a chosen threshold value are made external. One rationale behind this criterion is that the many reactions that contribute to mass balance of such a highly connected metabolite are reasonably represented by considering it as buffered, in a subnet not containing all those reactions. Another is that this choice is particularly effective at avoiding the combinatorial explosion problem. And finally, for a high enough threshold, mainly carrier and commodity metabolites are selected that are not the focus of interest in a typical subnet.

Using a threshold connectivity of 5, the metabolic network of *Mycoplasma pneumoniae *was found [13] to divide into 19 subnetworks, with identifiable biological functions. Similarly the human redox metabolism was found [14] to split into 7 subnetworks for the threshold value of 5. Although successful in these relatively small metabolic networks, a criticism expressed by several authors [11,12,15] is that it relies totally on a local property, the degree of node connectivity, and takes no account of the global network structure. Compared to their method that uses the global "bow-tie" structure, Ma et al [15] found that while both methods classify most currency metabolites similarly, there are examples in their method of both internal metabolites with high connection degree and external metabolites with a low degree. It was also acknowledged in the original article [13] that the fully automated selection on connection degree alone can be improved by minor editing based on biochemical knowledge. Despite these reservations, connectivity selection is still implemented in the current version of the network analysis software application YANAsquare [16].

The network splitting procedure presented in this article aims to incorporate the insights outlined above. In addition it provides flexibility to interactively guide how the splitting proceeds, based on the purpose and biochemical knowledge of the user, within the limits set by the inherent network structure.

The formulation adopts internal/external reassignment as the splitting paradigm, but only uses the connectivity degree as a preliminary coarse filter to identify the most obvious external metabolites. This is optionally supplemented or refined by an explicit listing of metabolites that are/are not taken as external. The main algorithm uses random walks to explore long range network structure, in a similar way as MCL clustering [9]. However instead of the rigid automated cluster delineation produced by the "inflation" step of the MCL, the results are displayed to the user as a matrix that summarises network structure even for large networks in a powerful visual form. At the heart of the visualisation is a blocking transformation designed to express subtleties of the status of each node in relation to an underlying hierarchical clustering, in a way that resembles fuzzy clustering algorithms. Optimisation using linear programming delivers a small set of candidate externals, which the user can accept or reject and this process is repeated until acceptably small subnets are produced. In a final postprocessing step, externals that are not essential for the partitioning achieved, are reincorporated to ensure that the inevitable loss of mass balance information is kept to a minimum. The procedure described here has been implemented in a software application called Netsplitter, and is subsequently referred to as the "netsplitter algorithm".

## Methods

### General overview

Processing of a metabolic network consisting of an unordered list of chemical reactions specified in the standard way by a matrix of stoichiometric coefficients, proceeds through four computational stages:

1. Generating a matrix representation of the network connectivity structure from random walks, which expresses each internal metabolite as a distinct source or sink node in an associated directed acyclic graph ( DAG).

2. Using hierarchical clustering and a blocking transformation to rearrange the DAG matrix into latent blocks that express the underlying partially separated subnets.

3. Proposing prospective separator nodes for approval to the user, implementing the decision and recalculating the DAG with improved blocking, leading to the next round of separator selection.

4. Post-processing to consolidate subnets by reincorporating superfluous externals and to reconstitute a stoichiometry matrix specification of each subnet from the DAG matrix blocks.

Each of these stages is described in more detail in the following subsections, followed by introduction of a quantitative measure of effectiveness. Fuller justifications for some of the steps are supplied in a separate subsection at the end of Methods.

### Matrix representation of biochemical networks

#### Random walks and probability matrices

The procedure is based on representing the network as a matrix of probabilities that reflect random walks on a simple graph, similar to that used in the well-known Markov Clustering (MCL) algorithm [9]. However, since a metabolic network contains nodes of two distinct types (metabolite and reaction), the first task is to reduce the stoichiometry matrix (S-matrix) used conventionally to specify a metabolic network, to a probability matrix for a simple graph containing metabolite nodes only. For this step it suffices to treat the metabolic network as a bipartite graph, although it has been pointed out that metabolic networks are best considered hypergraphs [17].

For a simple graph, one starts from a probability matrix **P**_1 _where the elements in row *i *are the probabilities that a random walk starting from node *i *in the network will reach node *j *in a single step. For simplicity, equal probabilities are assigned to all links emanating from a particular node. To achieve this, one merely needs to normalise each row of the adjacency matrix **C **of the graph, by dividing each element by the sum of all elements in the row. The probability matrix **P**_N _for a random walk of *N *steps, is calculated by raising **P**_1 _to the *N*-th power. Walks of *n *≤ *N *steps, will be included in this provided that there is a non-zero probability for a random walk step to "stay put" on a node; in other words self-loops are included in the adjacency matrix, or in matrix terms a multiple of the identity matrix is added before the row normalisation is carried out.

If we start from a state where there is a single "random walker" on each node of the network at step 0, the probability associated with each walker has the value 1 for being localised on its starting node. Then **P**_1 _represents propagation of this probability to nearest neighbour nodes in step 1, and generally the potentiating of the matrix can be visualised as the flow of probability through the network after increasing numbers of steps. This is expressed in MCL terminology by referring to potentiating as the "expansion" operation.

As constructed, the matrix **P**_1 _has non-negative elements with a row sum = 1, which makes it an example of a *right stochastic matrix*. From the theory of stochastic matrices [18], it is known that raising a matrix of this type to consecutive powers converges to a matrix denoted as **P**_∞_. In practice, for metabolic networks numerical convergence to an approximation of **P**_∞ _is obtained for values of *N *in the low tens. A typical feature of this matrix is that many of its columns are zero vectors, implying that after a sufficient number of steps, the probability of finding a random walker on the node corresponding to that column approaches zero, regardless of the node from which the walker started at step 0. Such nodes therefore act as *sources*, while the remainder with non-zero columns are *sink *nodes. In a thought experiment with one walker starting from each node of the network at time 0, all walkers will congregate on the sink nodes after the number of steps needed for convergence. A binary version of **P**_∞ _, obtained by replacing all non-zero elements by 1, can be interpreted as the adjacency matrix of a new graph, containing the same nodes as the original network, but in which all links connect sources directly to sinks in a star-like configuration. This is formally described as a directed acyclic graph, and is directed irrespective of whether any links in the original network were directed. In what follows, either **P**_∞ _or its binary version is referred to as the DAG-matrix. Qualitatively the features described above are quite similar to those in the MCL, but note that in MCL terminology only the "expansion" operation (raising **P**_1 _to a power) is applied while the "inflation" operation that is key to the MCL, is not used. Consequently the DAG obtained here does not usually separate into disconnected clusters, and needs to be further manipulated by the algorithm to extract subnetworks.

The generalisation of the procedure outlined above to the bipartite metabolic network case, starts by defining two separate adjacency matrices **CR **and **RC**. For a network of *m *reactions and *n *metabolites, the S-matrix is (*n *× *m*) with rows representing metabolites and columns reactions. **CR **is similarly (*n *× *m*) and each row contains the adjacencies of a metabolite node that act as a substrate to each reaction column, while **RC **is (*m *× *n*) and gives the adjacencies of reaction products but with the roles of rows and columns reversed. Formally, these are constructed from **S **by the relations

(1.1) $$CR=\frac{1}{2}\left(\left|Sign(S)\right|-Sign(S)\right)\;;\;RC=\frac{1}{2}Transpose\left(\left|Sign(S)\right|+Sign(S)\right)$$

Here the Sign function takes the values -1, 0 or 1 and serves to ensure that **CR **and **RC **are nonnegative binary matrices. From these the probability matrix for the reduced metabolites-only network is calculated by

(1.2) $$P_{1}=RowNorm\left(RowNorm\left(CR\right)•RowNorm\left(RC\right)+0.25\;I\right)$$

Here the function RowNorm normalises each matrix row by a simple row sum, and converts the adjacency matrices to probability matrices. The summation implied in the matrix multiplication accumulates the probabilities for a random walk jump from metabolite node *i *to metabolite node *j*, over all reactions that connect them. Note that links in the metabolites-only network are directed in accordance with underlying reaction directions, whether these are reversible or not. In the added term, **I **represents an (*n *× *n*) identity matrix to account for selfloops, and its coefficient is chosen to scale their probabilities in accordance with typical values in a sparse network. The results for the DAG are found to be insensitive to the actual coefficient value. Unlike for a simple graph, the selfloops could not be introduced into the adjacency matrices because they are non-square and even if not, it would have destroyed the bipartite nature.

Calculation of the DAG matrix proceeds by straightforward iterative potentiation of **P**_1_, using convergence of the Frobenius norm of the matrix to within an absolute value of 10^-10 ^as the criterion.

#### Matrix implementation of partitioning

Reclassifying an internal node as external to produce network partitioning, is implemented by deleting the corresponding row from **CR **and column from **RC**. This implies that at any stage the DAG matrix only represents internal metabolites, and as this changes during the course of the partitioning the DAG is regularly updated. A detailed account of this implementation is given in the justifications section further below.

#### Preprocessing the DAG matrix

The first step in processing the DAG matrix is to sort its columns so all non-zero columns are collected on the left and rows sorted in the same order, then deleting the zero columns. In this way, by definition only sink nodes remain in the column sequence and rows are sorted with all sinks appearing first, followed by all source nodes.

To demonstrate the method, an example network consisting of 137 metabolites and 117 reactions is used in what follows. This network happens to be a subnet for flavonoid metabolism in *Arabidopsis thaliana *extracted from the Aracyc [19] database, but serves here merely as a realistic example of a small metabolic network. The specification of this network in SBML format is available as Additional File 1.

**Figure 1:**
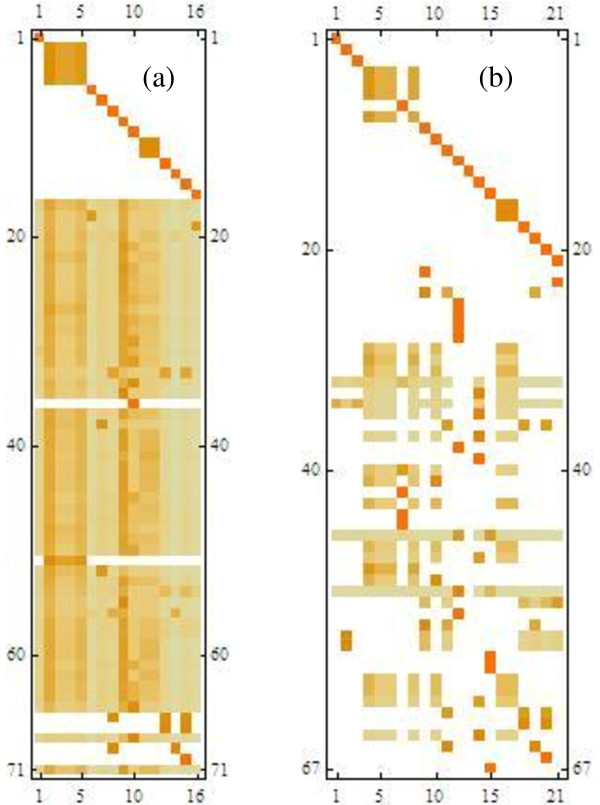
**DAG matrix for demo network**. Non-zero columns of the DAG matrix (a) with only structural externals recognised (b) after reclassifying 4 high connectivity internals as external. Colour scaling expresses random walk probabilities between source nodes (rows) and sink nodes (columns) of the network; comparison of (a) and (b) shows how connectivity structure is revealed by an appropriate high connectivity cutoff.

Figure 1(a) shows the DAG matrix for this network using a colour scale to represent numerical values of the matrix elements. Of the original 137 metabolites, 66 have been identified as a structural external (i.e. it acts uniquely as either a substrate or a product in all reactions in which it participates) and eliminated. The remaining 71 internals are found to separate into 16 sinks and 55 source nodes.

The top square 16 × 16 submatrix is seen to be (block) diagonal. For the majority of single diagonal elements, this merely indicates the finite probability that a random walk starting from a sink node will end there as a result of a selfloop, while it will not terminate at any other sink. There are also a few small blocks; they represent small clusters of nodes that are fully connected and hence jointly act as a "supersink". This top square does not reflect much of the overall network structure and further manipulation centers on the lower part, i.e. the DAG matrix is further truncated to contain just the (*nsources *× *nsinks*) lower left submatrix of the original.

Inspection of the lower 55 × 16 submatrix in Figure 1(a) shows very little internal structure on which to base network partitioning; almost all source nodes are connected to almost all sinks. The reason is that in a metabolic network there are typically a small number of ubiquitous metabolites such as carrier molecules that participate in many reactions. Sacrificing mass balance for these, is arguably not a serious loss of information, as an excess or lack of the molecule in the subnet can be assumed to be made up by other subnets. The common convention of presenting metabolic pathways as a backbone structure while suppressing secondary metabolites, is an implicit recognition that such crosslinking tends to obscure the underlying connectivity structure that is important to understand functional units in a metabolic network.

Reclassifying just the four highest connectivity internal metabolites (Water, Coenzyme A, NADP and NADPH) in the demonstration network produces the drastic change shown by Figure 1(b). The truncated DAG now exhibits clear structure, creating scope for further manipulation as described in the next section.

A useful strategy to determine such a set of a priori ubiquitous metabolites is to simply choose a fixed threshold value and reclassify all internal metabolites with connectivities higher than the threshold, in order to reveal the connection structure. A threshold of 8 was found to work well for networks over a wide range of sizes, from about 100 metabolites upwards. Manual adjustment of the threshold can also be done as its effect is easily monitored by visual inspection of the truncated DAG as in Figure 1.

Alternatively, an explicit list of commonly occurring ubiquitous metabolites can be used instead of a threshold, to avoid inadvertent reclassifications. The most efficient strategy was found to be a combination, using a threshold to automatically reclassify the most "obvious" carrier metabolites automatically, and supplementing this with an explicit list of less obvious ones.

### Rearranging the DAG matrix to identify subnets

#### Subnetworks and matrix blocks

The key insight needed to use the mathematical infrastructure described so far for network partitioning, is that separated subnets can be made to appear in the truncated DAG matrix as non-overlapping blocks.

A block is defined as a rectangular submatrix, formed by the intersection of a horizontal band of rows and a vertical band of columns, and where any non-zero matrix elements in either band occur only inside the intersection (so elements in the bands outside of the block are all zero). It follows that the row and column ranges of a block does not overlap with those of any other block. So if it exhibits a non-trivial block structure, the full set of rows in the truncated DAG matrix will be partitioned with no overlap into two or more bands, and similarly the columns into the same number of bands. This definition does not require that blocks are arranged diagonally.

The connection with disjoint subnets is established by noting that a non-zero element (*i*,*j*) in a particular block of the truncated DAG means that there is a finite probability, and hence a path through the network from source node *i *to sink node *j *in the same block. Conversely, the zero elements in the bands belonging to a particular block but outside that block, means that probability does not flow from source nodes in one block, to nodes in any other block; nor are sink nodes in one block fed by sources in any other block. So the collection of sources and sinks of each block, specifies the internal nodes of an isolated subnet.

The truncated DAG as constructed so far will not show such block structure, but two operations are available to produce the block structure:

• Rows and columns may be reordered. There is no penalty to this, as the ordering of internal metabolites inherited from the S-matrix is arbitrary.

• Internal metabolites may be reclassified as external and deleted from the adjacency matrices. This carries a penalty, as information is lost - the mass balance of the metabolite is not enforced any more. The DAG matrix needs to be recalculated in this case and usually has a different allocation of sinks and sources.

#### Rearrangement of rows and columns

The first step is to rearrange rows and columns so that metabolites belonging to a block are grouped together. For computational efficiency, operations described here are performed on a binary version of the truncated DAG matrix, on the grounds that it is the connectivity of the network that is relevant rather than detailed probabilities from the random walk. In a binary matrix with simple rectangular blocks, all rows/columns in a particular block are identical but are orthogonal to those in any other block. However, the definition of a block given previously allows zero elements inside a block as well, so this is relaxed to say that rows/columns belonging to a block needs to be similar to each other but dissimilar from those in other blocks - i.e., it reduces to a vector clustering problem. The Sokal-Sneath vector dissimilarity is used to quantify this, as discussed in more detail in the justifications section.

Using this measure the rearrangement problem reduces to one of finding row and column sequences that give optimal clustering. Of various standard clustering methods that were considered the hierarchical clustering method [20] was found to be most suitable.

Hierarchical clustering, as expressed in a dendrogram representation, has the advantage that - unlike most other clustering methods - it gives a definite sequence (the ordering of leaves in the dendrogram) while not committing to a fixed number or size of clusters. These can be subsequently determined by choosing a cutoff level in the dendrogram, a property exploited in the next stage of the procedure.

**Figure 2:**
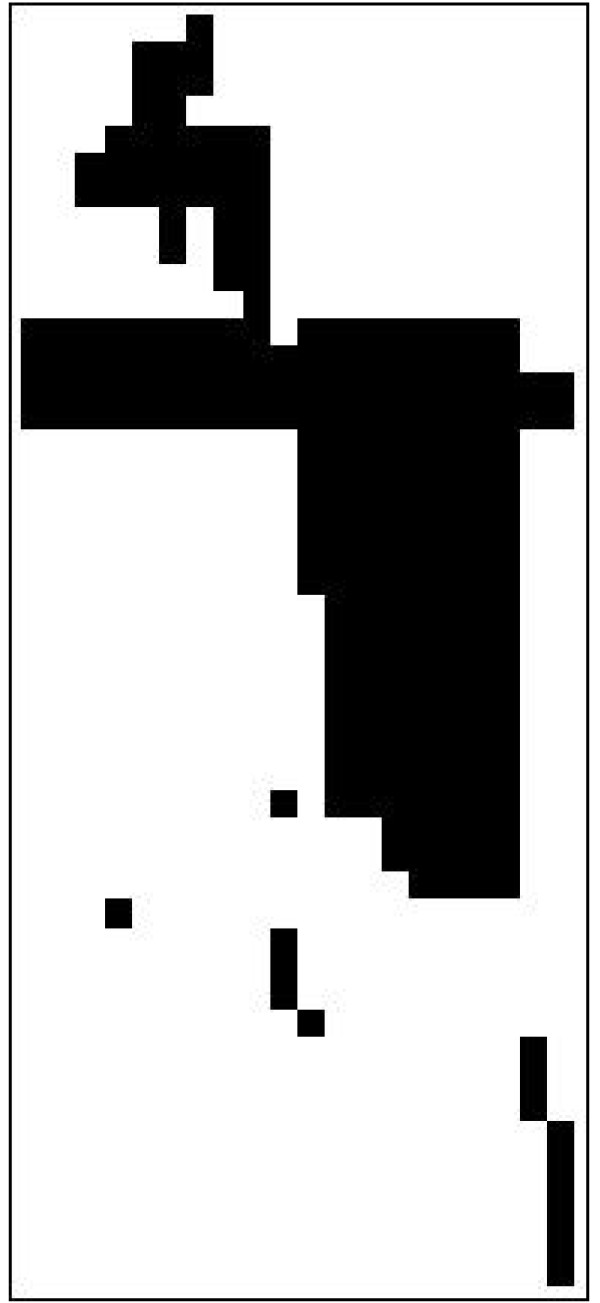
**Rearranged binary**. Binary truncated DAG matrix, reordered according to hierarchical clustering of rows and columns. Clustering groups nodes with similar long range connections together, so that black areas that form the cores of latent block structures appear.

Figure 2 shows the result of reordering rows and columns in the truncated binary version of the matrix of Figure 1(b) according to an agglomerative hierarchical clustering using single linkage and the Sokal-Sneath dissimilarity criterion. The figure shows a far better organised structure with black regions identifying associations between metabolites groups, but no separated blocks. This is expected - reordering alone can only isolate blocks if the underlying network (after removal of externals) was already divided into disjoint subnets.

#### Blocking transformation

The next challenge is to identify latent blocks that can be separated in further processing. A crucial decision to be made is the optimal number, size and shape of blocks. Reordering alone as in Figure 2, does not give any obvious clues to whether many small blocks or fewer large ones will best represent the network.

The decision is facilitated by introducing a *blocking transformation *that expresses the extent to which the hierarchical clusters succeed in defining blocks. The transformation proceeds in five steps:

1. Truncating the column dendrogram at a particular chosen level, defines a collection of consecutive column clusters such as *C *= (1-3,4-8,9,10-11,...). In the matrix, these clusters define vertical bands. In row *i *of the rearranged, truncated DAG matrix with elements *p*_ij_, we make the replacement

(1.3) $$p_{ij}\to r_{ij}=\frac{\sum_{j\in C_{j}}b_{ij}}{\sum_{j}b_{ij}}$$

Here *b*_ij _are the binary matrix elements and *C*_j _is the column cluster to which column *j *belongs.

In the case of a perfectly blocked matrix, all non-zero elements in a row will belong to the same unique cluster and their values are left unchanged at 1. Any zero element in the same cluster is replaced by 1, i.e. any gaps inside the cluster are filled in. All row elements in the remaining clusters will be, and remain, zero.

However, for an imperfectly blocked matrix, any non-zero element outside the range of a particular cluster will serve to dilute the common value of elements inside the cluster to a fractional value. Hence in a gray-scale representation the row appears as a sequence of bands in different shades of grey; the darkest grey identifies the cluster containing the largest fraction of non-zero elements.

**Figure 3:**
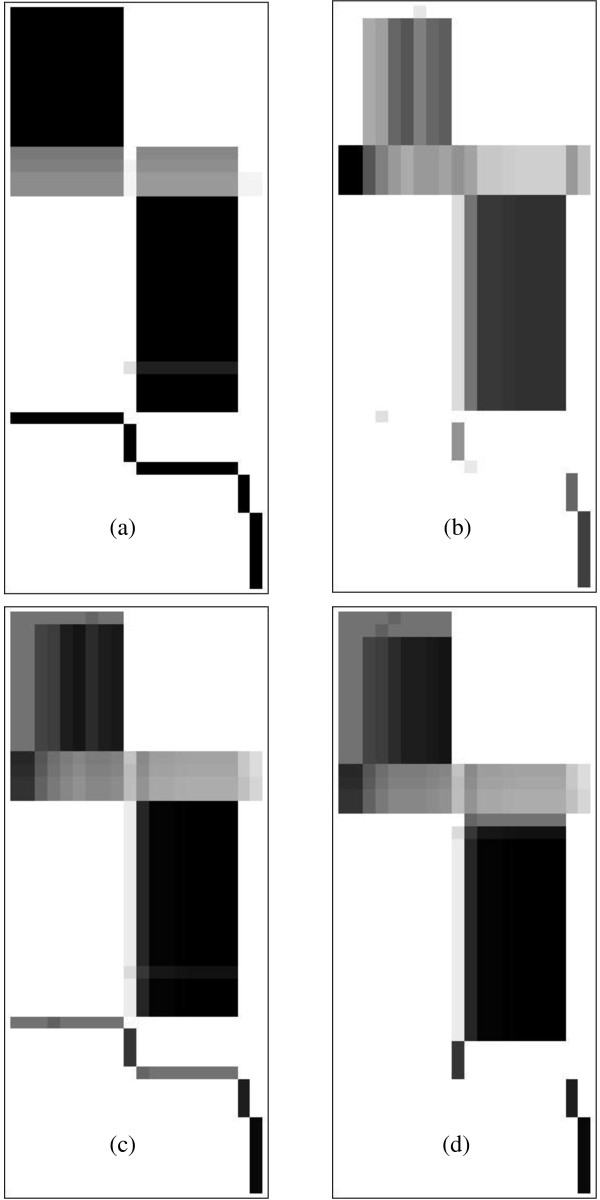
**Blocking matrices**. Transformed versions of the truncated binary matrix, constructed by (a) blocking rows (b) blocking columns (c) superimposing row and column blocking matrices (d) reordering rows and columns to consolidate blocks. Grey shades in effect expresses the degree to which rows conform to column grouping and vice versa. Combining these and optimising the clustering, expresses subnet cores visually as dark areas and overlaps by lighter shades.

Applying this transformation to all rows of the matrix in Figure 2 produces the row blocking matrix shown in Figure 3(a). Analogously, columns are blocked next:

2. Truncating the row dendrogram at a particular chosen level, defines a collection of consecutive row clusters *C*_i_. In column *j *of the rearranged, truncated DAG matrix, we make the replacement

(1.4) $$p_{ij}\to c_{ij}=\frac{\sum_{i\in C_{i}}b_{ij}}{\sum_{i}b_{ij}}$$

Application of this transformation to all columns similarly gives the column blocking matrix shown in Figure 3(b).

In a perfectly blocked matrix, blocks based on grouping rows or columns are identical, but the demonstration example shows that for imperfect blocking the row and column blocking matrices are similar but not identical. The next step superimposes the information from the two separate hierarchies.

3. Combine row and column blocking matrices by elementwise averaging:

(1.5) $$s_{ij}=\frac{1}{2}(r_{ij}+c_{ij})$$

The combined blocking matrix obtained in this way is shown in Figure 3(c). In this matrix, grey shades again indicate deviations from perfect blocking - whether by leakage of amplitude from hierarchical clusters or by discrepancies between row and column blocking. Black areas, on the other hand, identify areas where all evidence from the grouping procedures agrees and that can plausibly be taken as cores for blocks still to be further delineated. Moreover, the gap filling effect mentioned above serves to highlight the intrinsically rectangular shape of blocks.

An important aspect of the algorithm has been glossed over above. The dendrograms used in steps 1 and 2 define hierarchical lists of distances between subclusters. It is by choice of a particular cutoff value in each list (defining the minimal distance for subclusters to be recognised as separate) that one can choose between many smaller clusters or fewer larger ones.

To exploit that, a quantitative criterion *Q *for the optimal blocking has been defined as detailed in the justifications section, leading to the next step:

4. Calculate *Q *from equation (1.8) after repeating steps 1-3 for each of the trial list of cutoff values, and select the one that maximises Q.

The blocking matrices shown in Figure 3(a)-(c) are those that maximise Q for the demonstration network and represent the visual structure of Figure 2 quite well.

However, there is still one noticeable deficiency in Figure 3(c). Some blocks appear split - for example, there are two medium grey horizontal lines in the lower half of Figure 3(c) that each clearly belongs with the large dark areas directly above it. The reason for this is a conflict between similarity at the fine-grained (element) level used when grouping, and the coarse-grained (block) level that applies after blocking. This is rectified by the final step of the blocking transformation.

5. Consolidate blocks by reordering rows and columns according to hierarchical clustering now applied to the combined blocking matrix.

As Figure 3(d) shows, the final result of the blocking transformation gives a succinct and visually appealing overview of the size, shape and location of blocks, as well as indicating areas that produce block overlap.

Finally, in order to computationally process individual subnets, automated recognition of separated blocks is required. This is a fairly straightforward image processing problem, and a heuristic procedure based on the block definition given above is described in Additional file 2. A side effect of the heuristic is that matrix rows and columns are rearranged once more, aligning recognised blocks along the matrix diagonal.

### Selection of separation nodes

Having prepared the DAG matrix to express any underlying partial block structure, the procedure now enters an iterative loop in which in each round, a small number of nodes are identified that when "cut" (i.e., the corresponding metabolite is reclassified as external), will lead to separation into subnets. The goal is to keep this set of separation nodes as small as possible, both to minimise the loss of mass balance information and to preserve as far as possible the local structure of the full network.

For example, applying block recognition scanning to the matrix of Figure 3(d), recognises only the full matrix as a single block. That is also visually apparent - there are four or more latent blocks highlighted in black, but none are fully separated as signified by grey areas.

For a number of reasons, it is postulated that the lighter grey cells in the figure are the most promising candidates for removal to induce separation. One rationale is that by construction they reflect a status as exceptions while the majority of cells in their row or column belong to the same group and end up as dark grey. Also, they tend to result from cases where there is already separation from the perspective of the row grouping and only weak overlap from the column grouping, or vice versa. Middle grey, on the other hand reflects either strong evidence from one of the groupings, or moderate consensus that may solidify once the weakest overlaps are removed. Also, there is some analogy to the effects of the "inflation" step of the MCL. In that method, "inflation" is produced by taking the Hadamard power of a probability matrix; that tends to suppress low probabilities and leads to the "weakest" links between node clusters to be removed first. These arguments can be made more elaborate, but in the final analysis the justification lies in the result obtained. As detailed in the justifications section, linear programming is used to select a small number of metabolites that optimally cover the lighter grey cells in the blocking matrix and propose these to the user as candidate externals.

**Figure 4:**
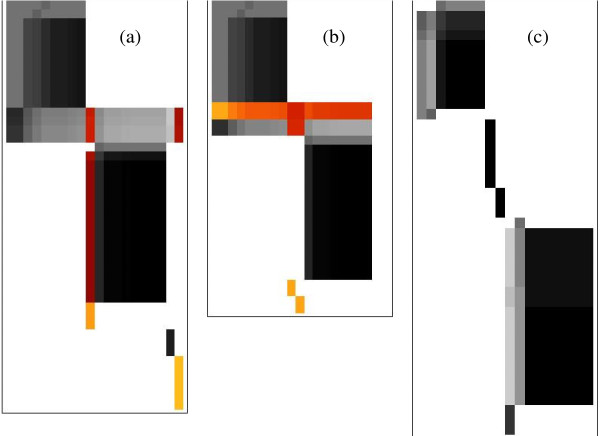
**Eliminating separation nodes**. Blocking matrices as presented to the user for selecting separation nodes in subsequent rounds. Rows and columns proposed for cutting are highlighted in colour. (a) First round (b) Round 5 (c) Final result, after seven rounds and restoring all blocks and reincorporation of superfluous externals. The four non-overlapping blocks represent separation into four subnetworks, the largest two still showing minor internal structure.

An example is illustrated in Figure 4(a) where this is applied to the case of the consolidated blocking matrix in Figure 3(d). The picture shows that two columns, i.e. metabolites that produce block overlaps (separation nodes), are identified by solving equation (1.9). These metabolites are made external, i.e. they are deleted from the adjacency matrices **RC **and **CR**, the DAG matrix recalculated and the blocking transformation repeated to identify further candidates in a second round, and this iteration is continued until either sufficiently fine-grained splitting has been achieved, or no more separation nodes are found.

Figure 4(b) shows the result in selection round 5. The blocking matrix still shows only one block, but has decreased in size, firstly because of the removal of separation nodes. Also, at each round the algorithm inspects the blocking matrix for any non-overlapping block that consists of a single row or column, like the two middle blocks shown in Figure 4(c). Such blocks cannot be split further, and are therefore removed from the blocking process to be restored later.

Another case of such irreducible blocks that is usually encountered, is the appearance in the DAG matrix of isolated sinks or "orphans". These appear as entries in the top, diagonal section of the DAG matrix with no accompanying source node entries in the corresponding column of the truncated matrix used for blocking. Such an orphan metabolite node signifies the simplest possible subnet, with only a single internal metabolite, and typically containing only two reactions. As these can obviously not be further split and the presence of an empty column complicates block recognition, they are best eliminated in each round from the adjacency matrices along with single row/column irreducible blocks.

### Postprocessing and reconstruction of subnetworks

Once the iterative process of progressively selecting separation nodes has terminated, the main outcome is a list of internal metabolites, partitioned into disjoint subsets that belong to each block. The remaining metabolites constitute a list of external metabolites. This list may contain entries that are not, in fact, essential for block separation. For example, a metabolite may have been made external during initialisation on the grounds that it participates in a large number of reactions, but if all of those reactions belong to the same subnet it should be reinstated as an internal metabolite in this subnet. Also, it can happen that the effects of a metabolite selected early on in the progressive selection process, are superseded by one selected later. In the interests of maintaining maximal network integrity compatible with the separation, all such superfluous externals need to be reincorporated before finalising the subnets.

This is done in a loop that inspects the stoichiometry matrix for each external metabolite on the list, to determine all internal metabolites to which it connects by reaction links in either direction in the bipartite representation. If all those belong to a single block, the external metabolite is reincorporated into that block. If they belong to a single block, except for a connection to one or more orphan metabolite nodes, those orphan nodes are also reincorporated into the block as detailed below. As this reincorporation loop changes the composition of the lists of internal and external metabolites, the loop is repeated iteratively until there is no further change in the composition of the lists.

The approach that was chosen to select separation nodes progressively, a few at a time, has the advantage that it allows the user to steer the network splitting by accepting or rejecting proposed separation nodes and terminating the process at the desired level of granularity. However, a disadvantage is that the results may become dependent on the order in which separation nodes are identified. That is counteracted by performing a one-off blocking step in which the full list of external metabolites are applied simultaneously. This step is performed as part of the post-processing done after the selection process is finished; but the question arises whether it should be done before or after the reincorporation step. Each choice has some advantages, and the most robust result is in fact achieved by repeating the reincorporation step. So the full post-processing procedure consists of the 3-step sequence: a first reincorporation step, then the one-off blocking, followed by a second reincorporation. Figure 4(c) shows the effects of the reincorporation for the demonstration network.

Once the partitioned list of internal metabolites is finalised by this post-processing, the individual subnets can be reconstructed in a straightforward way from the original stoichiometry matrix **S**. For each subnet, all reactions in which its internal metabolites participate are extracted from **S **and allocated to this subnet. All metabolites that participate in these reactions are collected; those not appearing on the list of internals for the subnet, are by definition the external metabolites of the subnet. The submatrix of **S **pertaining to the reactions and metabolites so identified is extracted and saved in appropriate format as a full specification of the subnetwork, which can be further analysed by standard network analysis or FBA software tools.

By construction, the internal metabolites of different subnets are mutually exclusive sets. External metabolites, on the other hand, are often shared between subnets. In the vast majority of cases, there is also no overlap between external metabolites of any subnet and the internals of any other.

There are, however, rare exceptions where an external of one subnet is in fact an internal of another. This phenomenon can be considered an artefact of the way that the algorithm mainly operates on a reduced metabolites-only simple graph. At this level where the blocking procedure is carried out, there is a strict distinction between internal and external metabolites; they form non-overlapping sets. However, when translated back to the underlying bipartite graph representation, cutting all metabolite nodes that were identified as external, can sometimes still leave subnets connected by a shared reaction node.

**Figure 5:**
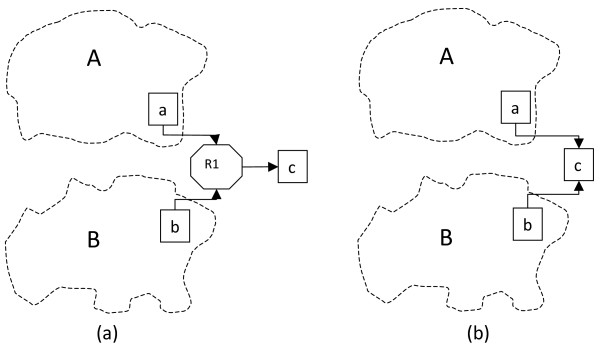
**Example of internal-external overlap between subnets**. Subnets A and B, connected by a common product of reaction R1. Metabolite nodes are shown as squares and the reaction node as an octagon. (a) Bipartite representation of the network (b) Reduced metabolites only network. In (b) subnets are fully separated by making *c *external, but in (a) reaction R1 is included in subnet A to ensure full representation of the network environment of internal node *a *.Hence node *b *acts as an external for subnet A, but as internal for subnet B.

A typical case is shown in Figure 5(a), where subnets A and B are connected by reaction node R1 which has input reactants *a *and *b *respectively from each subnet. Figure 5(b) shows the metabolites-only representation, where splitting node *c *by making metabolite *c *external will separate the subnets. But then, after the blocking procedure, the external metabolites of each subnet are found by collecting metabolites that participate in all the reactions in which each internal metabolite is involved. In the case of subnet A, both nodes *b *and *c *are connected to its internal node *a *by reaction R1 and will be added to its list of externals; and conversely, for subnet B, metabolite *b *is internal while *a *and *c *become externals.

The existence of this kind of limited overlap between two subnets does not compromise the integrity of either as a coherent subnet: it remains true that for all internal metabolites in a subnet, all reactions in which they participate are included in the subnet, and so the mass conservation constraints of all internal metabolites are identical in the subnet and in the full network. However, it does uniquely create the complication that the same reaction is present in both subnets, which can lead to conflicting values for the flux through this reaction in separate FBA calculations for each subnet. To avoid that, it may be preferred to merge the two subnets into a larger one when this exceptional case arises.

It should also be noted that for a similar reason the reincorporation of orphan metabolite nodes is slightly more complicated than outlined above. By definition, an orphan node is isolated from all other internal nodes in terms of probability flow, but it could still be connected by a unidirectional link towards the orphan. Consequently, incorporation of an orphan takes place in two steps. When an external connected to an orphan metabolite node is incorporated into a block, the orphan is first promoted to an external of that block. In the next round of the incorporation loop, it is then tested for links to internals of other blocks and only incorporated as an internal if no such links in either direction is found.

### Detailed justifications

#### Internal and external metabolites and network partitioning

Conventionally, external nodes are placed on the periphery when drawing a network to indicate that they form the interface between the metabolic system that the network represents and its environment. However, the distinction between nodes that are associated with mass balance constraints (internal metabolites), and those that are not (external metabolites) is not apparent when the network topology is simply specified as a list of reactions. Most external metabolites can be recognised computationally by the fact that an external metabolite is either taken up or delivered to the environment so that all network links impinging on an external node are directed away from or towards the node; but in cases where the metabolite is exchanged with the environment that distinction is lost.

A convention commonly used in FBA of metabolic networks [1] to keep track of externals, is to order the rows of the S-matrix so that internal metabolites appear first and externals last. Then the lack of stoichiometry constraints for externals is easily implemented by using only the top (internals) section of the matrix for FBA calculations.

Another feature of representing a chemical network by a bipartite graph, is that as reaction nodes represent a chemical transformation of one or more reactants, reaction nodes can never be external.

These issues become relevant for partitioning a network, because in isolating a subnetwork a new periphery is created for it. Severing the connection between the subnet and the rest of the network, some metabolites are received from or/and delivered to the rest of the network. Their mass balance can no longer be guaranteed by the subnet alone; in other words, the status of these metabolites is changed from internal to external. From a graph theory perspective, partitioning corresponds most naturally to deleting a link of a graph. However, that will not do for the biochemical network; in the bipartite representation, that would make a reaction node external, and it makes even less sense in the metabolites-only simple graph representation where a link represents a sum over several reactions. In clustering methods such as MCL, each node is allocated to a particular cluster, but that would not make sense here either as a metabolite that is made external by partitioning belongs to both subnets - as a product of one, and substrate of the other subnet. Clearly the appropriate way to represent partitioning is to split the metabolite node into two, each becoming an external node in either subnet. This leaves all reaction nodes as internal and uniquely assigned to a subnet. The effect of splitting a node is to stop probability flow through the node, and the simplest way to implement that in the matrix representation, is to delete the corresponding row from **P**_1 _and hence ultimately from the DAG.

The problem of partitioning the network hence reduces to finding a suitable (by criteria to be formulated) subset of internal metabolites such that when deleted, the network divides into self-contained subnets with no probability flow between them.

Recognising that the algorithmically found externals are due to be deleted in this way, it follows that metabolites that are already external in the full network should similarly be deleted from **P**_1 _even before the partitioning starts. This step in fact corresponds to the restriction of FBA calculations to the internal rows of the S-matrix as mentioned before. However, in the procedure presented here rows are deleted from the adjacency matrices **CR **and **RC **while **S **is left intact, so that the reaction stoichiometries can be used to restore the externals to each subnet once partitioning is complete.

#### Vector clustering

A quantitative measure of dissimilarity *d *for binary vectors can be based on the formula

(1.6) $$d=\frac{n_{10}+n_{01}}{n_{10}+n_{01}+a\;n_{11}+b\;n_{00}}$$

Here, corresponding elements in two equal length binary vectors are paired, and *n*_ij _is the number of pairs with value (*i*,*j*). Commonly used such measures are known as Matching (*a *= 1, *b *= 1), Jaccard (*a *= 1, *b *= 0), Sokal-Sneath (*a *= 1/2, *b *= 0), Rogers-Tanimoto (*a *= 1/2, *b *= 1/2) and Dice (*a *= 2, *b *= 0) dissimilarities. Experimentation with all of these in the present context has shown that Matching and Rogers-Tanimoto give low contrast. Dice, Jaccard and Sokal-Sneath are similar but the best contrast and hence block identification is obtained with Sokal-Sneath dissimilarity.

Hierarchical clustering in addition needs a measure for the distance between clusters (a "linkage criterion"), and again several common measures were tried: single linkage (minimum intercluster dissimilarity), complete (maximum intercluster dissimilarity), average dissimilarity, dissimilarity of cluster centroids or medians, and finally the Ward minimum variance criterion. Single linkage was found to be both fast to calculate and gives good contrast; average, centroid and median are slower but give similar results, while complete and Ward lead to excessive fragmentation of the network.

Clustering of the combined blocking matrix in step 5 of the blocking transformation is performed broadly as described for the DAG. However since the combined blocking matrix can contain fractional values, the binary dissimilarity measure described by equation (1.6) is replaced by a generalisation of the Dice dissimilarity to real values and known as the Bray-Curtis distance between vectors *a *and *b*:

(1.7) $$d=\frac{\sum \left|a_{i}-b_{i}\right|}{\sum \left|a_{i}+b_{i}\right|}$$

#### Blocking quality

Experimentation with various possibilities yielded the following scoring formula for the blocking quality *Q *of an (I×J) combined blocking matrix:

(1.8) $$Q={\left(\frac{\sum_{i,j}c_{ij}}{I\;J-W}\right)}^{2}\frac{W}{I\;J}$$

Here, W is the total number of zero (white) elements in the matrix. The two factors in this formula express distinct features that seem qualitatively reasonable to judge the quality of the blocking matrix. The first, squared, factor would be 1 if all non-zero elements are 1 (black) and decreases when there are more and lighter grey cells; so it is a measure of "how black" the block parts of the matrix is. On its own, however, maximising this tends to favour a small number of large blocks because that makes it easier to capture all the non-zero values inside blocks. To counteract that, the second factor represents the fraction of cells that are white, so this tends to be maximised by keeping blocks as compact as possible. It clear that for a perfectly blocked matrix, a maximum *Q *value *Q*_max _< 1 will be achieved if the cutoff produces clustering that coincides exactly with the blocks.

In an imperfectly blocked matrix, adjusting the dendrogram cutoff gives unpredictable fluctuations in the *Q *value and so a search method is necessary to maximise *Q*. Three strategies are employed to keep this manageable. First, as the number of recognised clusters only changes when the cutoff rises above an actual intercluster distance value in the dendrogram, the search space is restricted to a trial list of discrete cutoffs falling midway between values in the ordered intercluster distance list. Second, the same cutoff value is applied to both the row and column dendrograms. Although it constrains the flexibility of the search, this helps to avoid large disparities in the number of clusters for rows and columns (which ideally should be equal) despite the fact that the truncated DAG matrix generally has far more rows than columns. To implement that, the intercluster distance lists for rows and columns are merged before selecting midpoints. Finally, bearing in mind that distance values from equation (1.6) fall between 0 and 1, the discrete list of trial cutoffs is truncated to values in the range [0.2,0.7]. This avoids either very few or very many blocks that would not be desirable for subnet partitioning and rarely produce high *Q *scores anyway.

#### Optimal selection of separator nodes

To implement the recognition of light grey cells in the blocking matrix as most promising for eliminating block overlap while keeping the metabolites taken as external to a minimum, the strategy is to select the smallest set of rows and columns that together cover all matrix cells with values below a chosen threshold. The threshold is determined as the value that selects a total number of light grey cells, no more than a low multiple of the column dimension of the matrix. This gives a flexible threshold value adapted to the size and nature of the matrix, which will lead to only a few metabolites eliminated at a time before checking for adequate subnet separation.

As any light grey cell could be eliminated by taking either its row or column metabolite external, the optimal selection from both sets is determined by reformulating this as an integer linear programming (ILP) problem. To set that up mathematically, introduce a binary column vector **x **of dimension = number of internal metabolites. Each vector element is 1 or 0 according to whether the corresponding metabolite is selected. The total number selected is obtained by premultiplying **x **with a row vector **b **of the same dimension with all elements equal to 1. The constraints are that for each light grey element *s*_ij _included, either its row or column or both needs to be present in **x**. That is codified by a constraints matrix **A **in which each row corresponds to a light grey cell, and in such a row the only non-zero elements are 1's for the columns corresponding to *i *and *j*. Then the ILP problem is:

(1.9) Minimize b⋅x subject to A⋅x≥b

This problem is to be solved in the domain of binary vectors, and is guaranteed to be feasible, since all constraints are satisfied by **x **= **b**. Solution by standard methods typically yields small sets of selected metabolites.

### A quantitative measure of overall splitting effectiveness

The goal of subnet splitting is to reduce the complexity of interpretation (mentally or by further computation) by reducing the size of networks that need to be considered. It is shown here that a robust quantitative measure of how effective a particular splitting procedure is in achieving this goal can be developed under quite general assumptions.

The original network constitutes the obvious lower limit of simplification. In the opposite extreme where the network is fragmented into subnets consisting of a single node each, no overall simplification has been achieved either: while the subnets are simple, their interconnections reconstitute exactly the original network. This suggests that to judge overall effectiveness, the subnets should be considered together with a "metanetwork" which is derived from the original network by contracting the internal nodes of each subnetwork to a single meta-node. It should then be possible to construct a measure that evaluates to zero at the two extremes, and reaches at least one maximum at a suitable intermediate network partitioning.

To quantify the concept of simplification, it useful to introduce a monotonically increasing function *f*(*n*) that represents the effort to interpret a network with *n *internal nodes. Having split a network with *N *internal nodes into *k *subnets, each with *n_i _*internal nodes, the metanet has *k *nodes and the total effort *F *to interpret the metanetwork and all the subnets is then given by

(1.10) $$F(\vec{n},k)=f(k)+W\overset{k}{\underset{i=1}{\sum }}\;f(n_{i})$$

and this is subject to the constraint $\sum n_{i}=N$. With a weight *W *= 1, equation (1.10) represents the effort to interpret the metanet and all subnets; choosing *W *= 1/*k *is relevant to the case where a single subnet and its interconnections to other subnets are of interest. The latter case is algebraically simplest so is considered first.

The term "effort" is used to emphasize that this is not about network complexity as such. Many sophisticated measures of network or graph complexity have been defined by various authors, and network size usually does not play an important part in this - for example, both a square lattice and a fully connected network are conceptually simple, irrespective of size. Also, biochemical networks are known to be scale-free (having a power law distribution of node connectivity) and so complexity measures should give a similar value when applied to the full network and its subnetworks.

For a given *k*, it follows by straightforward differentiation of equation (1.10) that the constrained minimisation of *F *is achieved by choosing all subnets of equal size, i.e. *n*_i _= *N*/*k *, provided that *f*(*n*) is concave up, i.e. increases monotonically faster than *n*. This is quite reasonable, considering that the number of reaction links in a metabolic network rises approximately quadratically with the number of internal metabolites and the number of pathways to be interpreted much faster than that.

Moreover, setting *n*_i _= *N*/*k *into (1.10) shows that the minimum is achieved at the optimal number of subnets *k *= $\sqrt{N}$, irrespective of the functional form of *f*. This result makes intuitive sense, as it implies that all subnets and the metanet have the same number *n_i _*= $\sqrt{N}$ of internal nodes, so the interpretative effort is spread equally across them all. The value of *F *at its minimum is given by the simple formula

(1.11) $$F_{l}(N)=2f\left(\sqrt{N}\right)$$

When $\sqrt{N}$ is not integer, this minimum cannot actually be reached, but it still serves as a lower limit to the range of *F*, while the upper limit is obviously *F*_u_(*N*) = *f*(*N*). In these terms the goal of partitioning can be formulated as finding a vector $\vec{n}=(n_{1},n_{2},...n_{k})$ that comes as close as possible to *F_l_*, having started from *F*_u_. This leads to defining a performance measure (designated as the *efficacy*) as

(1.12) $$E=100\frac{Log\left[f(N)\right]-Log\left[F\left(\vec{n},k\right)\right]}{Log\left[f(N)\right]-Log\left[2f\left(\sqrt{N}\right)\right]}$$

This may be interpreted as the percentage of the distance between the upper and lower limits that has been achieved by a given partitioning, as measured on a logarithmic plot. Use of logarithmic scaling is not conceptually essential but helps to smooth the distribution of efficacy values when *f *is a fast-rising function.

It is easily checked that *E *evaluates to zero for the original network and could even reach a slightly negative value for the case of complete fragmentation, while it gives 100% if the optimum *k *= *n*_i _= $\sqrt{N}$ is reached.

To get concrete values, a power law assumption produces the required concave up behaviour while still allowing the actual rate of increase to be adjusted:

(1.13) $$f(N)=\alpha N^{p}\;;\;p>1$$

A value *p *= 2 to reflect the size of the adjacency matrix that fully specifies the network seems reasonable, but the choice is left open for now. The proportionality constant *α *cancels out in constructing (1.12) so is suppressed in what follows.

**Figure 6:**
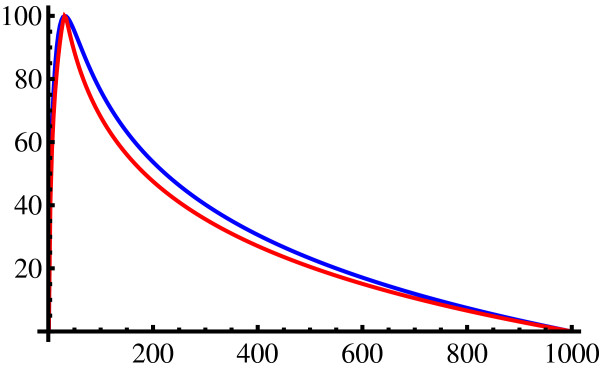
**Efficacy curves**. Efficacy % as function of subnet count *k *for equal size subnets from a network with *N *= 1000 internal nodes. Top (blue) curve is for a power law with *p *= 1.5, lower (red) curve for *p *= 10. The curve peaks sharply at *k *= $\sqrt{N}$ but is insensitive to *p*.

The efficacy curves calculated from equations (1.12) and (1.13) for equal-sized subnets, at subnet counts *k *ranging from 1 to *N*, are shown in Figure 6 for *p *= 1.5 and *p *= 10. With a smooth variation and quite a steep gradient, in particular near the optimal subnet count, *E *gives a sensitive measure of how well a particular network split performs in terms of its subnet count. The figure also shows that this behaviour is almost independent of the *p*-value, with higher values merely sharpening the maximum slightly.

The main significance of the *p*-value is in determining the discrimination between partitionings with the same *k*, but homogeneous versus distributed subnet sizes. Raising the power value increases the dominance of larger subnets over smaller ones in the summation term of equation (1.10), which tends to lower the efficacy value for a split with a large spectrum of subnet sizes.. Experimenting with various networks has shown that for large networks *p *values in the range 6 to 8 are required to adequately discriminate between network splits that are dominated by large subnets, and those with more even size distributions. For small networks the available range of subnet sizes is correspondingly small and the value of *p *less important. The best results are obtained by adjusting *p *to the network size, and the following empirical formula performs this adequately:

(1.14) $$p=0.25\sqrt{N}$$

This formula is merely a calibration of the efficacy scale and has no fundamental significance. The results below illustrate its effects.

It is finally noted that the efficacy measure is constructed quite independently of the Netsplitter method; as its only required input is a list of subnet sizes, it can equally well be applied to diverse partitioning algorithms.

## Results

The results obtained from the Netsplitter procedure are illustrated by considering the problem of investigating the flavonoid metabolism of the model plant *Arabidopsis thaliana*. The complete network was obtained by extracting all stoichiometrically balanced reactions from the Aracyc 4.5 database as curated by the Arabidopsis Information Resource (TAIR) [19] and contains 1468 reactions and 1348 metabolites. Reaction directions and reversibilities were assigned in accordance with the pathways and enzymes tables of the database. It is not intended as a definitive metabolic network for the organism (no further curation e.g. using flux balance calculations was done) but for the demonstration purpose it is considered adequately representative of genome scale metabolic networks. The specification of this network in SBML format is available as Additional File 3.

**Figure 7:**
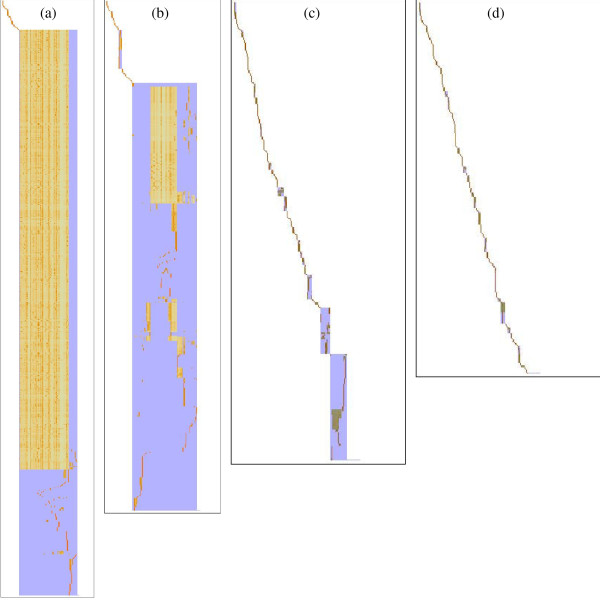
**Matrix visualisation of simple connection degree network partitioning**. DAG matrix for genome-scale network of *Arabidopsis thaliana *after applying connection degree partitioning with different cutoffs. Subnetworks are displayed as non-overlapping blocks (coloured pixels on blue background) for connectivity threshold value (a) 20 (b) 10 (c) 5 and (d) 4. Large values leave a monolithic unresolved block, while small cutoffs produce extensive fragmentation.

For comparison, Figure 7 first shows the performance of the simple connection degree partitioning [13] for various threshold values. Visual representation of the DAG matrix is as in Figure 1, but elaborated here by the addition of a blue background that displays automatically recognised block limits.

For a large threshold value of 20, Figure 7(a) shows that only a few small blocks are split off from the main block that still contains more than 90% of all internal metabolites. As observed in the demonstration example, internal structure in this large block is not resolved. Reducing the cutoff threshold to a value of 5 used in previous work [13,14], the internal structure is well resolved but the main block still contains 20% of all metabolites. Reducing the threshold still further to 4 finally avoids the domination of a single large block, but at the cost of fragmenting the network into 164 very small blocks containing only 4 internal metabolites on average.

**Figure 8:**
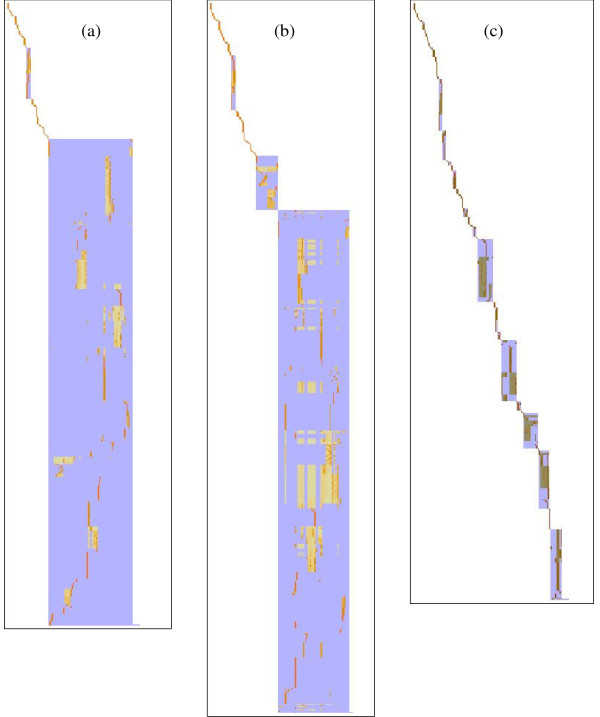
**Stages in partitioning the network by the netsplitter procedure**. *Arabidopsis thaliana *network (a) After application of connectivity threshold 10 and external metabolite list only (b) After first round of separation node selection (c) Final result after 29 selection rounds. Most of the network is partitioned into medium sized to small subnets, with some fragmentation remaining.

Figure 8 shows that the netsplitter algorithm achieves a much more even distribution of subnet sizes. Using the same initial threshold value of 10 shown in Figure 7(b) but supplementing this with a targeted list of general external metabolites as specified in Additional File 4, has the effect shown in Figure 8(a) of resolving the internal structure of the initial main block. The netsplitter algorithm exploits this internal structure to split it progressively. In its first round, a medium sized block which turns out to contain all flavonoid compounds is split off as seen in Figure 8(b). For the purpose outlined above the procedure can be terminated at this point, and this subnet extracted for further study as was done to create the flavonoid demonstration network used above. However, if the procedure is allowed to run its course, the final result is shown in Figure 8(c). There is a substantial improvement, in that rather than a single large block, there are several medium sized ones. Quantitatively, 66% of all internal metabolites are captured in the 10 largest blocks which range in size between 20 and 76 internal metabolites. There is still some fragmentation, with the remaining 34% spread over 68 small blocks containing 3.6 metabolites on average. Some fragmentation is probably inevitable when making cuts to a network, but future work will be aimed at reducing this to a minimum.

The reincorporation step is important to keep the number of externals as low as possible. For example, in the full *Arabidopsis *network, 17 of the 57 high connectivity externals and 12 of the 22 orphan metabolite nodes created by taking a connectivity degree threshold of 10, can be reincorporated without disturbing the block structure in Figure 8(a). When the netsplitter algorithm is subsequently applied, these numbers are reduced because of the increased partitioning achieved, but in turn 112 of the 150 externals proposed during the course of externals selection rounds are in fact reincorporated in the final stage. Even in the case of the small flavonoid network where only a single separation round is needed producing 8 proposed externals, 6 of these get reincorporated leaving only two separation nodes. The overall effect is that in both cases less than 5% of the significant mass balance constraints in the full network are sacrificed to decompose it into subnetworks.

**Figure 9:**
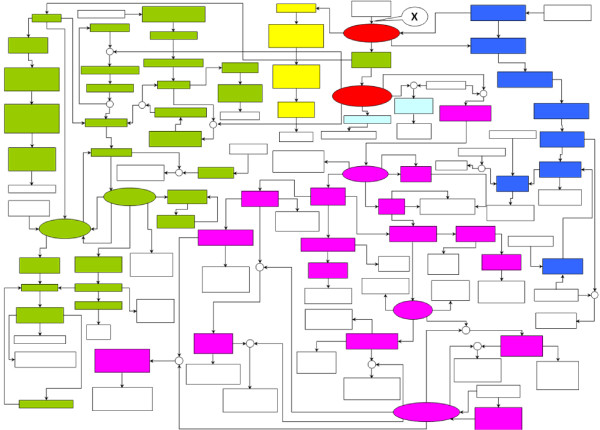
**Example flavonoid network split into four subnetworks**. Simplified layout omitting commodity and currency metabolites, to show partitioning into 6 subnetworks by converting the two separator metabolites identified by Netsplitter from internal to external. Reactions are shown as arrows or small circles. Metabolites are shown as rectangles or ovals, colour coded as follows: white - external; yellow, green, blue, purple: subnetwork internals; red - separation nodes, light blue - orphan metabolite nodes. The reaction indicated by "X" is eliminated from the network because after conversion it only involves external metabolites. A fully labelled version of this figure is available in Additional File 5.

It is also instructive to see the action of the netsplitter procedure in an explicit network diagram. The actual layout of the flavonoid demonstration network, for which stages in the procedure were traced out in matrix form in Figure 1 to 4, is shown in Figure 9. A larger version of this figure identifying the metabolites is available as Additional File 5.

The algorithm identifies two separation nodes in this case - the metabolites trans-cinnamate and coumaroyl-CoA (shown in red); cutting these, the network falls apart into four natural subnets, plus two small fragments or "orphans". By inspection of the metabolite names (not shown in the figure) the subnets can be identified as synthesis of flavonoids (purple), lignin precursors (green), benzenoids (blue) and coumarin (yellow). While in this relatively small network it may have been possible (although not easy) to identify these separators by inspection, it should be borne in mind that much of the work to group nodes coherently has already been done in the manual construction of the two-dimensional layout displayed. In a realistic example, the input to the algorithm is merely arbitrarily ordered lists of metabolites and reactions, making the task much harder.

To place the results in a more general perspective, Table 1 compares efficacy values for various cutoff values in simple connectivity-based partitioning [13,14,16], with Netsplitter results applied to four different networks of increasing size. In addition to the flavonoid demonstration network and the genome scale model plant *Arabidopsis *discussed in detail above, genome scale networks for a simple bacterial species and a mammal are included. The first of these is a metabolic model for *M. pneumoniae *recently published by Yus et al [21]. Details of the application of Netsplitter to this model has been published elsewhere [22] and is shown there to give a partitioning that is virtually identical to the assignment by Yus et al of pathways to functional blocks, based on biochemical knowledge. The second is a model for *Mus musculus *validated by FBA [23].

**Table 1:** Efficacy values

	Flavonoid	*M. pneumon*	*Arabid. t*.	*M. musculus*
S-matrix size	137 × 117	189 × 229	1468 × 1348	2016 × 2158

*p*-value	2.1	2.8	7.2	7.6

Threshold	*High connectivity cutoff method*			
20	0	33	18	25
10	19	33	24	32
8	19	33	27	37
6	31	37	49	**41**
5	**80**	45	**54**	33
4	76	**60**	43	27
3	29	32	32	21

Netsplitter	*Default automated externals and reincorporation*			
	88	85	70	48

Values are shown as percentages, and peak values highlighted in bold. The p-value increases with network size as described in the text.

Considering first the connectivity based splitting, Table 1 illustrates that as expected, lower efficacies are calculated for large connectivity cutoffs where a large part of the network remains undivided, and also for small cutoffs where the network is fragmented. This produces an intermediate maximal efficacy, at cutoff values close to 5, in accordance with the values chosen empirically [13,14] on similar small networks. The p-values assigned by equation (1.14) fall well within the range between the curves in Figure 6 in all cases.

In judging efficacy percentages, its increased sensitivity near the optimum as illustrated in Figure 6 should be borne in mind - e.g., a partitioning with an efficacy *E *= 80% is about six times closer to the optimal subnet size than one with *E *= 40%.

**Figure 10:**
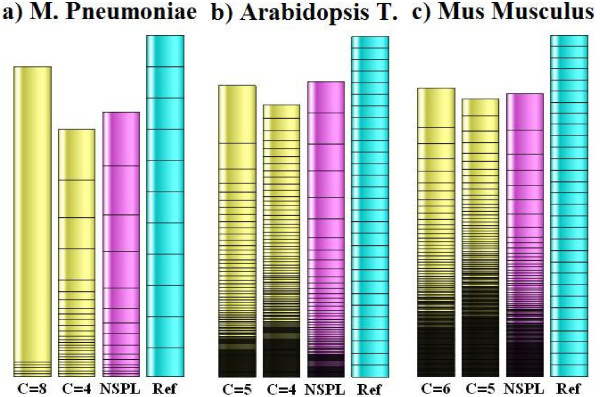
**Stacked bar chart representation of network splits**. Subnet sizes for different partitioning of genome size networks of three organisms. Yellow bars represent connectivity partitioning with the indicated cutoff values C, magenta bars the Netsplitter partitioning followed by a cyan reference bar that shows theoretical maximal efficacy partitioning of the original network. In each bar, each subnet is represented by a segment with height proportional to subnet size, and subnets have been sorted in order of increasing size towards the top. Fragmentation is indicated by dense stacking at the bottom. Decreasing the cutoff to split the monolithic bar at the top increases fragmentation, but Netsplitter results improves both aspects.

While a single numerical score can hardly be expected to capture all the varied considerations (some subjective) of what constitutes the best partitioning, the more detailed graphical representation in Figure 10 of the most interesting cases, suggests that *E *as calculated here does give a good overall quality indication.

In that figure, each bar segment corresponds to a subnet and the total height of each bar represents the total number of internal metabolites for that partitioning. Thus the height difference from the reference bar on the right, indicates the total number of internal metabolite mass balance constraints that have been sacrificed to achieve a particular split. The reference bar also shows the theoretical maximal efficacy $\sqrt{N}$ size partitioning of the original network. As this totally ignores the network topology, no actual partitioning can be expected to achieve that. But a reasonable aim would be for subnets to have this size on average without too large deviations on either side.

Figure 10(a) illustrates that a large connectivity cutoff for the bacterial network (the first bar) gives the opposite effect, with one large block and a few very small ones, and this has a correspondingly low efficacy value of 33%. The cutoff *C *= 4 in this case splits the large block into medium sized ones approximating the reference value, but at the price of considerably increasing both the number of small blocks and the number of lost constraints, while achieving an efficacy of 60%. The Netsplitter result improves on both aspects and gives *E *= 85%.

In the case of *Arabidopsis*, the *C *= 5 value that maximises *E *gives a couple of rather large blocks and many small ones, including fragments too small to be resolved graphically and appearing as black or grey areas at the bottom. Decreasing the cutoff to 4 worsens the fragmentation, and this is reflected in the *E*-values of 54% and 43% respectively. It is again visually obvious that the Netsplitter result improves on both of these by spanning the reference subnet size more effectively, and has a moderately high score of *E *= 70%. It has less fragmentation than either of the cutoff results, and it also retains more constraints than either.

A rather similar situation is shown in Figure 10(c) for the case of *M. musculus*, The best cutoff value increases slightly to *C *= 6 for this larger network, but already shows large fragmentation reflected in *E *= 41% and again this worsens both visually and in terms of the calculated efficacy to *E *= 33% if *C *is reduced to 5. Visually, the Netsplitter result has a slightly better spread of sizes at the top and somewhat better but still significant fragmentation at the bottom, giving an only moderately improved efficacy score of 48%.

In all cases, the efficacy score based on equation (1.14) accords quite well with observations from the more detailed graphical display.

The analysis above of the performance of the netsplitter algorithm for the larger networks, shows that there is a decline with size but that this is not due to its efficiency in splitting, but rather that fragmentation becomes an increasing problem as networks grow. A direct approach to solve that is to introduce controlled merging of subnets and this will be further explored in a subsequent article.

## Discussion

Previous work [24] has shown that graph theory algorithms to trace pathways through a network can give results in conflict with stoichiometric constraints. Netsplitter is not of this kind; graph analysis is merely used to identify a list of external metabolites, but network splitting is done directly on the stoichiometry matrix and (apart from the reclassifications) all the constraints that it expresses are maintained intact. Nevertheless, the concern may exist that e.g. elementary modes may be lost through the partitioning.

A general counterargument is that removal of constraints cannot reduce the number of solutions to a problem. More specifically, consider for example a single mode that in the full network traverses two of the subsequent subnets. When the subnets are separated by reclassifying the metabolite node on their interface as external, the mode is correspondingly split into two parts. Since the original mode satisfied all constraints set by mass balances of internal compounds along its path, the two parts must separately continue to be viable. One part will now belong to the first subnet and terminate at the boundary node, which has become an unconstrained external sink and cannot affect its viability. The other will start at the corresponding unconstrained boundary source node in the second subnet and similarly remain viable because by construction the network context of internal metabolites nodes in each subnet is identical to that in the full network.

A direct demonstration of this is obtained from a comparison of the null space of the internal stoichiometry matrix in the full network and in the subnetworks. As the flux vector of a mode lies in the null space, a reaction can only be active (i.e., participate in any of the modes) if there is a non-zero entry in at least one basis vector of the null space. For example, in the flavonoid demonstration network shown in Figure 9, there are 117 reactions, and calculation of the null space basis shows that 116 of those are active. Repeating this calculation for the internal stoichiometry matrix of each of the six subnets gives (42, 17, 5, 47, 2, 2) active reactions respectively in each subnet. As reactions are uniquely allocated to subnets in this case, the counts can be added to give a total of 115 reactions that are active collectively in the subnets. That leaves a discrepancy of one reaction, the one indicated by an "X" at the top of Figure 9. This reaction becomes excluded from the flux space because reclassification of the separator metabolite trans-cinnamate (indicated as the red oval node nearest the top) implies that after partitioning the reaction becomes irrelevant as it then only involves external metabolites. The single inactive reaction in the full network, ends up in subnet 4 and remains inactive there.

Performing this null space analysis for genome scale networks such as those shown in Figure 10 reveals the same behaviour in all cases. Reclassification can result in some reactions involving only external metabolites and consequent reduction of the flux space; but in the remainder, all reactions that are active in the full network remain active in the subnets, and inactive ones remain inactive in subnets as well. This confirms that subnets collectively support the same set of modes as the full network.

The reduction of the flux space is another perspective on the desirability of keeping the set of external metabolites as small as possible, as is implemented in Netsplitter. Nevertheless, it is observed that the reactions eliminated are (like the one in Figure 9) mostly those at the periphery that only involve a single internal metabolite in the full network, and that their removal has a minimal effect on the structure of modes.

Computing efficiency has been taken into account in several aspects of the Netsplitter procedure. Performing the main computation on a metabolites only simple graph rather than the bipartite representation, reduces matrix dimensions roughly by a factor of two, since the numbers of metabolites and reactions are usually similar. As about half of the metabolites are typically external, including only internals gives a further dimension reduction by a factor of about two. Focussing on the (*nsources *x *nsinks*) submatrix of the DAG gives a further reduction by a similar factor, enhanced also by using binary representations for clustering. Bearing in mind that matrix manipulations usually scale quadratically or worse, the overall reduction in complexity could approach two orders of magnitude.

This is borne out by moderate computing times. The total running time observed for the demonstration network of 117 reactions × 137 metabolites is 1.25 seconds, while for a genome scale network of 2037 reactions and 2179 metabolites this increased to 59 seconds, on a Core 2 Duo PC with 4 Gb of memory running at 2.66 GHz. These values appear quite acceptable and indicate that the algorithm scales better than quadratic with network dimension. It may be possible to achieve better performance by rewriting the code in a compiled language, but as extensive use is made of sophisticated graph theory and user interface functions built into *Mathematica*, this would be a major undertaking. Experimentation with *Mathematica *options to compile the most computing-intensive sections of the code did not produce significant performance improvements, suggesting that the coding of these functions is already quite efficient.

The procedure as presented is quite elaborate and requires considerable programming for its implementation. To facilitate its practical use, a software application "Netsplitter" has been developed as a *Mathematica *[25] notebook which is available for download [26]. This application provides an interactive interface that displays the progress of the subnet separation in the way illustrated by Figure 4, and offers additional facilities not discussed here such as the merging of selected subnets and display of subnet layouts. A more complete description of the software aspects and illustrations of its application to large scale networks, will be presented in a subsequent article.

An intriguing observation made in applying Netsplitter, is the radical change in resolving network structure that results from excluding high connectivity metabolites. An example is seen by comparing Figure 1(a) and 1(b).

It is surmised that the reason for this behaviour can be understood from percolation theory [27]. In networks where only a random subset of the potential links in an infinite regular lattice are occupied (i.e., present in the actual network), it is found that there is a critical occupancy of potential links, termed the percolation threshold *p*_c_. Long range paths that penetrate the entire network only exist for occupancies greater than *p*_c _. Values [28] of *p*_c _for a variety of lattices have been derived mathematically or numerically; for a simple infinite 2-dimensional square lattice this is 0.5 and typical values range between 0.3 and 0.6, including some non-regular or randomized lattices, while lower values are obtained in higher dimensions. The values also depend strongly on the coordination number *z *(i.e. the number of links impinging on a node). While a metabolic network is not a regular lattice nor necessarily 2-dimensional, a rough estimate of the occupancy can be made from the adjacency matrices by the formula

(1.15) $$p=\frac{\sum CR\cdot RC}{\;z\;N}$$

where a simple sum over all matrix elements is taken, and N is the total number of nodes. Applying this to the example network, it is found that the removal of the four high connectivity metabolites produces a sharp drop in the value of the occupancy from a value of 0.630 for the network in Figure 1(a) to 0.295 for Figure 1(b). This is consistent with the interpretation that the disappearance of long-range connectivity displayed by the figure, is caused by a drop of the occupancy number below an unknown percolation threshold. The same phenomenon has been observed in several other networks including those of genome-scale size discussed above.

Based on this understanding, an automated strategy could be pursued to progressively reclassify the highest connectivity internal metabolites as external until there is a sharp drop in the *p *value calculated from equation (1.15). In practice, however, it works equally well to simply choose a fixed threshold value and reclassify all internal metabolites with connectivities higher than the threshold.

Regarding the proposed efficacy measure, it was indicated above that it relies mainly on a general framework and even where a particular functional form such as the power law in equations (1.13) and (1.14) was postulated, its parameters merely readjust relative sensitivities to detailed features of the partitioning. This was further tested by experimenting with different functional forms such as an exponential dependence instead of a power law, taking *W *= 1 as might seem more plausible in equation (1.10), and even replacing the arithmetic mean term in that equation by a geometric mean expression. These changes were found to have marked effects on the complexity of the analysis, some of which could as a result only be done numerically. In the end the results were quite similar (but in some cases inferior in terms of smoothness and stability) and the simple option presented is deemed adequate.

The efficacy score measures the degree of simplification achieved by a given network partitioning. As shown in its derivation this is mathematically maximised for equal sized subnets. That does not mean that equal sized subnets is the ideal partitioning outcome; simplification is not the only criterion by which to judge success. Clearly there would be no special functional or biological relevance to an equal sized partitioning. On the other hand the low efficacy opposite extreme (towards which simple degree-based partitioning tends for large networks) of a large monolithic block and small fragments, or even complete fragmentation, is also functionally meaningless. As the *M. Pneumoniae *example illustrates, good agreement with conventional pathway assignments accompanies a moderately high efficacy value. In this sense, despite the inherent limitations of a single number score to represent varied considerations for judging the success of splitting a network for a particular purpose, efficacy values are useful as an overall guideline. It is noted that the efficacy measure is only used after the fact, optimising it does not form any part of the Netsplitter algorithm.

## Conclusions

The modularization of a large, complex biochemical network into subnets that can be associated with recognisable biological functions, can be helpful both in the conceptual understanding and interpretation of the network, and to reduce practical problems that arise in the application of analysis methods such as constraint-based modelling. The challenge in constructing an algorithm for this task is to accommodate both the objective structural properties of the network, and more subjective requirements such as the desire for a manageable number of subnets of roughly similar sizes. Also, while it is inevitable that some information will be lost when a subnet is isolated from its larger context, it is desirable to restrict this loss to information that is not subjectively relevant for a particular study.

In the procedure proposed here, dealing with the information aspect is facilitated by selecting metabolite node cutting as the partitioning operation, since this pinpoints the nature of the information loss as removal of a mass balance constraint. Then the subjective requirements are met by allowing the flexibility to veto the selection of particular nodes to be cut, or to terminate partitioning at a suitable subnet size. Both local and long range network structure is taken into account by the use of random walks and clustering strategies, and finally information loss is minimised by using optimisation techniques in selecting candidate separation nodes and by explicit reincorporation of nodes not essential for the separation.

The combination of these strategies succeeds in moderating the extremes of the subnet size distribution that results from partitioning based simply on connectivity degree.

This point is illustrated by considering Figure 7(c) and 8(c) as alternative outcomes for somewhat similar levels of intervention with Figure 7(b) as a common starting point. Figure 7(c) shows how simply decreasing the connection degree threshold from 10 to 5, introduces 61 new externals and increases the block count by 70, but most of these are very small and a large block encompassing 20% of the network remains. In Figure 8(c) the netsplitter algorithm incurs a similar but smaller information loss of 52 new externals, but only forms 30 additional blocks and encapsulates 66% of the network in 10 medium sized blocks. This is clearly a much better cost:benefit ratio, although it is recognised that parts of the network are still dispersed into small fragments leaving room for future improvements.

The efficacy measure *E *that was introduced encapsulates these considerations into a single quantitative quality score.

At present, it seems that the most promising applications of subnet splitting would be to studies and interpretation of network structure, such as those based on elementary mode analysis, rather than for the more quantitative FBA. In this context, subnetworks can play an important role in reducing the often very large number of elementary modes in a large network. The use of subnets for FBA would similarly simplify the problem and allow the elimination of extraneous detail not relevant for study of a particular aspect of metabolism. However, the obstacle that arises is that it would usually be more difficult to fix the boundary conditions ( i.e. flux values for metabolite exchange with the environment) for a subnet than for the full network. At least for a single cell organism, full network boundary fluxes reflect overall nutrient uptake or waste elimination rates that are relatively easy to measure. Externals of a subnet are likely to include metabolites shared with another subnet and measuring the associated fluxes may require much more detailed metabolic measurements. In special cases, such as when the subnet is spatially localised e.g. to a particular cellular organelle, this might present less of a problem.

A by-product of the matrix oriented approach used by the netsplitter algorithm, is the visually powerful display of network structure. Even for large networks for which a network layout diagram is totally unintelligible, features of network connectivity can be recognised at a glance from the colourscale plot of the truncated DAG matrix.

Even more striking is the characterisation of fully and partially resolved subnetworks afforded by grayscale plots of the blocking matrices. The blocking transformation that was introduced as the basis for computational recognition and optimisation of blocks and their overlaps, serves this second purpose to visualise rather subtle structural network properties. Quite apart from the purpose to separate subnets, this visualisation should be a useful tool e.g. to explore the structure of large networks or to compare how related networks differ from one another.

## Competing interests

The author declares that they have no competing interests.

## Supplementary Material

Additional file 1 **Demonstration model**. Specification of the network model used for demonstration in the Methods section, as an SBML file. Click here for file

Additional file 2 **Heuristic for block recognition**. A description of the heuristic employed by Netsplitter for automated recognition of non-overlapping matrix blocks as defined in the text. Click here for file

Additional file 3 **Genome scale Arabidopsis model**. Specification of the network model extracted from Aracyc 4.5 and used for demonstration in the Methods section, as an SBML file. Click here for file

Additional file 4 **External Metabolites**. Listing of default external metabolites, specified as Biocyc compound ID's. Click here for file

Additional file 5 **Demonstration network layout**. The network layout shown in Figure 8, with all metabolite and reaction nodes labelled with their Biocyc ID's and names. Click here for file
