# Supplementary material for: A new computational method to split large biochemical networks into coherent subnets
Source: BMC Syst Biol. 2011 Feb 7;5:25. doi: 10.1186/1752-0509-5-25 (PMC3045323; doi:10.1186/1752-0509-5-25)
Supplement: Additional file 2 — Heuristic for block recognition. A description of the heuristic employed by Netsplitter for automated recognition of non-overlapping matrix blocks as defined in the text. [file 1752-0509-5-25-S2.DOC]

# Heuristic for delineating matrix blocks

The procedure used for automated recognition of non-overlapping blocks in the Netsplitter procure is described below. It is based on the following operational definition of a block, taken from the article:

“A block is defined as a rectangular submatrix, formed by the intersection of a horizontal band of rows and a vertical band of columns, and where any non-zero matrix elements in either band occur only inside the intersection (so elements in the bands outside of the block are all zero). “

It follows that the row and column ranges of a block does not overlap with those of any other block. So if it exhibits a non-trivial block structure, the full set of rows in the matrix will be partitioned with no overlap into two or more bands, and similarly the columns into the same number of bands.

This definition does not require that blocks are initially arranged diagonally. The following heuristic detects such non-diagonal blocks, reorders matrix rows and columns such that blocks become aligned along the diagonal, and outputs the row- and column-index partitioning lists that specify block boundaries in the matrix. These partitioning lists can e.g. be used to visually display recognised blocks by a coloured background in a numerical or colourscale representation of the matrix.

First, rows are scanned as follows. Starting with the first row, the columns are divided into 3 consecutive (possibly empty) ranges such that all non-zero matrix elements fall in the middle range. The same is done for the next row, and if their middle ranges overlap the ranges are merged. This continues downwards until a row is found for which the middle range does not overlap with the cumulative one from above. This is recognised as a block boundary in the vertical direction, so the row range covered so far is stored as the row range for the first horizontal band, and the process restarted while scanning down further. When the last row is reached, a partitioning of both rows and columns into subranges will have been accomplished, which is easily seen to define the block boundaries in any perfectly blocked matrix.

In an imperfectly blocked matrix, horizontal scanning as described can be prevented from distinguishing column ranges by minor overlaps. An easily visualised example is a square matrix with two diagonally arranged blocks, made to overlap by a single stray pixel in the upper right “white” submatrix. In this case, resolution of the two column ranges would be accomplished by doing the horizontal scanning upwards starting from the bottom row.

An alternative is to use column oriented or vertical scanning in a similar way to produce a set of column and row subranges and this works better in some situations.

The following heuristic was found to be robust and produce excellent block recognition in all applications tried so far, including full genome-sized matrices. First, upwards and downwards horizontal scans are done, and the one that produces the partitioning into the largest number of subranges is retained. Then, vertical scans from both left and right are done and again the largest partitioning retained. Next, the horizontal and vertical partitionings are compared.

For any single row and column partition where the subranges derived from horizontal and vertical scanning are identical, this guarantees that the conditions for an isolated block are satisfied. Rows and columns in the matrix are reordered to move these “found” blocks to the beginning of their respective sequences. Where not identical, the vertical and horizontal subranges are inspected for cases that can be made identical by merging neighbouring ranges in one of the two sets. This ensures that small blocks that overlap only in one direction are correctly taken as one larger block and this is again moved to the beginning of the sequence. The process is repeated, because it is found that by moving found blocks out of the way, recognition of other blocks are facilitated. Iteration is continued until no more change is observed.

A fortuitous side effect of the reordering produced by the scanning heuristic, is that now all blocks are arranged in sequence along the main diagonal of the matrix.
